# Osteoarthritis Increases the Risk of Dementia: A Nationwide Cohort Study in Taiwan

**DOI:** 10.1038/srep10145

**Published:** 2015-05-18

**Authors:** Shih-Wei Huang, Wei-Te Wang, Lin-Chung Chou, Chun-De Liao, Tsan-Hon Liou, Hui-Wen Lin

**Affiliations:** 1 Department of Physical Medicine and Rehabilitation, Shuang Ho Hospital, Taipei Medical University, Taipei, Taiwan; 2 Department of Physical Medicine and Rehabilitation, School of Medicine, College of Medicine, Taipei Medical University, Taiwan; 3 Graduate Institute of Epidemiology and Preventive Medicine, National Taiwan University, Taipei, Taiwan; 4 Department of Physical Medicine and Rehabilitation, Changhua Christian Hospital, Changhua, Taiwan; 5 Graduate Institute of Injury Prevention, Taipei Medical University, Taipei, Taiwan; 6 Department of Mathematics, Soochow University, Taipei, Taiwan; 7 Evidence-Based Medicine Center, Wan Fang Hospital, Taipei Medical University, Taipei, Taiwan

## Abstract

Osteoarthritis (OA) and dementia are prevalent causes of disability in geriatric patients. To date, information on the temporal correlation between these progressive diseases and the risk of dementia in patients with OA is limited. This retrospective population-based 4-year cohort study investigated the risk of dementia in patients with OA. We performed a case-control matched analysis by using the Taiwan Longitudinal Health Insurance Database 2005. Patients were selected on the basis of International Classification of Diseases, Ninth Revision, Clinical Modification codes for OA between January 1, 2004 and December 31, 2007. The prevalence and the adjusted hazard ratio (HR) of dementia in patients with and without OA were estimated. The OA cohort comprised 35,149 patients and the non-OA cohort (comparison cohort) comprised 70,298 patients (1:2). The incidence of dementia was 21.7 per 10,000 person-years in the OA cohort and 14.7 per 10,000 person-years in the non-OA cohort. The HR for dementia during the follow-up period was 1.33 (95% confidence interval [CI], 1.17−1.50, P < 0.001) for patients with OA. The adjusted HR for dementia was 1.25 (95% CI, 1.10−1.43, P < 0.001) for patients with OA. The results of this study indicated that OA is an independent risk factor for dementia.

Osteoarthritis (OA) of the knee is one of the leading causes of disability among noninstitutionalized elderly adults^1^. Consequences of severe knee OA, including loss of mobility and limited daily activities, affect individuals and society economically. The World Health Organization (WHO) Global Burden of Disease Study, conducted in 21 epidemiological regions worldwide, reported a 26.6% increase in the burden of knee OA from 1990 to 2010^2^. Although nonsteroidal antiinflammatory therapy, exercise, and control of body weight remain the primary treatment options, there is no effective treatment for severe knee and hip OA, and total joint replacement is typically the final and only effective solution for relieving pain and disability.

Dementia, an age-related disease characterised by a gradual decline in mental abilities that affects reasoning, memory, and other cognitive functions, is one of the major causes of mortality in patients with knee and hip OA^3^,^4^. The incidence of dementia is 10% in people aged older than 65 years and approximately 20% in people aged older than 80 years. The prevalence of dementia in people older than 65 years was estimated to be 7.29% in Western Europe, 5.65% in South Asia, 8.50% in Latin America, and 4.98% in East Asia^5^. The prevalence of neurodegenerative disorders is increasing because of changes in the population demographics. As of 2010, 35.6 million people worldwide were diagnosed with dementia, and the numbers were estimated to nearly double every 20 years, to 65.7 million in 2030 and 115.4 million in 2050^5^. Health care costs can impose a substantial economic burden on patients with dementia^6^. Moreover, dementia causes a decline in cognitive and social functioning that interferes with independent living, affecting the individual and increasing the burden on family members and caregivers^7^. Therefore, preventing dementia in geriatric patients is crucial.

The correlation between OA and dementia has not been investigated thoroughly. An animal study reported that OA can accelerate the progression of and exacerbate Alzheimer’s disease (AD) by causing peripheral inflammation, triggering neuroinflammation, and inducing AD pathogenesis^8^. A previous study reported that primary total knee replacement can improve not only the knee function but also the mental health of patients with OA and mental disability. We hypothesized that OA can be an accelerating factor in the development of dementia. However, information regarding the occurrence of dementia after the onset of OA is limited. No large-scale nationwide population study has investigated OA and the risk of dementia. Therefore, we conducted a retrospective population-based case-controlled cohort study to investigate the risk of dementia among patients with OA.

## Methods

### Study design and study population

#### Data source

This retrospective population-based cohort study was conducted using case-control matched analysis. The patient data were obtained from the Taiwan Longitudinal Health Insurance Database 2005 (LHID2005). In March 1995, the National Health Insurance programme was established in Taiwan, and currently has over 25 million enrolees, covering more than 99% of the population of Taiwan. The LHID2005 comprises claims data for 1,000,000 beneficiaries randomly sampled from the Registry for Beneficiaries of the National Health Insurance Research Database (NHIRD), which contains 25.68 million claims files. The claims files contain information on ambulatory care; inpatient care; pharmacy use; date of service; International Classification of Diseases, Ninth Revision, Clinical Modification (ICD-9-CM) diagnostic codes; and claimed medical expenses. The National Health Research Institutes (NHRI) manages the claims data and provides scrambled random identification numbers for insured patients to protect patient privacy. Because the NHIRD comprises deidentified secondary data that were analysed anonymously, the need for informed consent in this study was waived.

### Study patients

Between January 1, 2004 and December 31, 2007, patients whose ambulatory care claims contained ICD-9-CM code 715 (OA) were identified. For an accurate diagnosis of OA, patients who received at least 5 consistent diagnoses according to the ICD-9-CM in outpatient clinics or a primary diagnosis of OA during hospitalisation within 1 year were selected. Initially, 35,901 OA patients were enrolled in this study. However, 450 patients were excluded because of missing data, and 302 OA patients with a previous sustained diagnosis of dementia (ICD-9-CM code 290) were also excluded. The OA cohort comprised 35,149 patients with OA, and the non-OA cohort comprised 70,298 patients without OA who were matched with the patients with OA in a 1:2 ratio according to age and sex. Each patient was monitored for as many as 4 years, from the patient’s entry date until the patient received 2 consecutive diagnoses of dementia (ICD-9-CM codes 290, 294, and 331) in outpatient clinics or 1 primary diagnosis during hospitalisation, or until the end of 2011. The database consists of deidentified secondary data, and the data were analyzed anonymously; therefore, the need for informed consent was waived.

### Baseline comorbidities

Baseline variables, namely age, diabetes mellitus (DM; ICD-9-CM codes 250 and 251), hypertension (ICD-9-CM codes 401−405), hyperlipidaemia (ICD-9-CM codes 272.0−272.4), autoimmune disease (rheumatoid arthritis [RA], ICD-9-CM code 714.0; systemic lupus erythematosus [SLE], ICD-9-CM code 710.0), cerebral vascular accident (CVA; ICD-9-CM codes 430−438), chronic obstructive pulmonary disease (COPD; ICD-9-CM code 496), and Parkinson’s disease (ICD-9-CM code 332), were obtained for all patients.

### Statistical analysis

First, we calculated the dementia hazard function for as many as 4 years by using the Cox model to examine the differences in the risk of dementia between the 2 OA and non-OA cohorts, after adjusting for patient physical characteristics, namely age and sex; comorbidities, namely DM, hyperlipidaemia, RA, CVA, COPD, and Parkinson’s disease; and the external factor of urbanisation level. To adjust for the hospital cluster random effect, we proposed a frailty model for modelling corrections among the dementia diagnoses of hospital clusters, incorporating a random component for the hazard function.

### Let the hazard function for the *i*th patient in the *k*th hospital cluster be


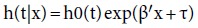
, where h0(t) is the baseline hazard function, x is the vector of the fixed-effect covariance (including OA, age, sex, autoimmune disease, DM, hypertension, hyperlipidaemia, CVA, COPD, and Parkinson’s disease), β is the regression coefficient, andτ is the random effect for the hospital cluster. Let τ ~ iidN(0,Δ). The penalised partial log likelihood is


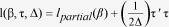
, where *l*_*partial*_(*β*) is the partial likelihood function for the Cox model. Thus, the marginal log likelihood of this shared frailty model is


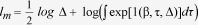


Using a Laplace approximation, we computed the optimal linear unbiased predictors of β,τ and Δ by maximising the penalised partial log likelihood^9^,^10^,^11^. All data analyses were performed using the SAS statistical package (Version 9.1.3; SAS Institute, Cary, NC, USA). A P value of <0.05 was considered statistically significant.

## Results

**Figure 1:**
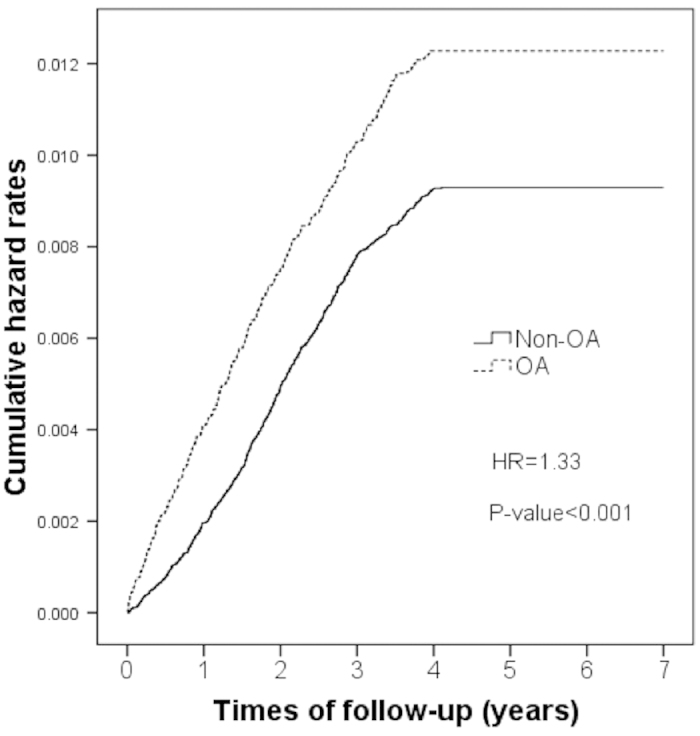
Kaplan–Meier hazard curve for dementia in osteoarthritis (OA) patients and the controls over a 4-year follow-up period.

The OA cohort comprised 35,149 patients with OA, and the non-OA cohort comprised 70,298 patients without OA. Women constituted 63.2% in both cohorts, and the prevalence of comorbidities was higher in the OA cohort than in the non-OA cohort (Table 1). The incidence of dementia was 21.7 per 10,000 person-years in the OA cohort, whereas it was 14.7 per 10,000 person-years in the non-OA cohort. In patients with OA, the crude hazard ratio (HR) for developing dementia was 1.33 (P < 0.001), and the adjusted HR was 1.25 (P < 0.001; Table 2). Figure 1 shows the Kaplan–Meier hazard curves for dementia in the OA and non-OA cohorts over a 4-year follow-up period. The log-rank test analysis in Fig 1 revealed that patients in the OA cohort had higher hazard rates (HR = 1.24, P < 0.001) than did patients in the non-OA cohort. According to the Cox regression analysis, the HR for dementia during the follow-up period was 1.32 (95% confidence interval [CI], 1.17−1.49) for the OA cohort compared with the non-OA cohort. After adjustment for patient age and sex, DM, hyperlipidaemia, hypertension, coronary heart disease, COPD, stroke, and Parkinson’s disease in hospital or clinics, the HR for dementia during the 4-year follow-up period was 1.22 (95% CI, 1.07−1.38) for patients in the OA cohort (Table 3).

## Discussion

Our study revealed that OA patients are at a high risk for dementia. This longitudinal cohort study is the first large-scale population-based study to investigate the risk of dementia in patients with OA. A community cohort study of geriatric patients with chronic, painful hip or knee OA reported that uncontrolled symptoms can cause functional disability and fatigue, which in turn lead to a depressed mood and ADL dependence^12^. In addition, patients with OA exhibit a high coprevalence of other chronic diseases. Among patients with OA, 90% have at least one additional chronic disease, of which cardiovascular diseases are the most common. Weight-bearing activities such as walking can exacerbate the pain and reduce the activity level of patients with OA^13^. A lack of physical activity may result in poor fitness and a higher risk of cardiovascular disease^14^,^15^. Nuesch *et al*. studied the causes of mortality in patients with OA and reported high mortality rates for all disease-specific causes of death, particularly for deaths associated with cardiovascular diseases and dementia^3^.

Previous studies have reported the preventive effects of physical activity and exercise on dementia later in later^16^,^17^,^18^,^19^. We suggest that improving physical activity after joint replacement is crucial in preventing dementia, particularly in geriatric patients with OA. Pain relief and restoration of ambulation function can enable patients to achieve higher levels of physical activity. A prospective cohort study revealed a strong correlation between total daily activity and a lower risk of dementia^20^. In addition, obesity is a key risk factor for OA, as indicated by the high prevalence of hip and knee OA in overweight patients^21^. Furthermore, a recent population-based study indicated that high midlife leisure and physical activity levels protect against dementia and AD, particularly in overweight people^22^. Studies have suggested that physical activity reduces cardiovascular risk by controlling blood pressure and exerting positive effects on brain structures^21^,^23^,^24^,^25^. Conversely, discontinuing daily activities because of OA can expose patients with OA to a higher risk of dementia.

OA is a slow degenerative disease, and the pathogenesis of OA includes loss of articular cartilage with subchondral bone remodelling. Proinflammatory cytokines induce inflammation, which is one of the pathological mechanisms of OA development^26^. Interleukin-1 (IL-1) and tumour necrosis factor (TNF alpha) are the key proinflammatory cytokines that induce cartilage catabolism in patients with OA^26^,^27^,^28^. Similarly, peripheral inflammation may be associated with an increased risk of dementia. In addition, previous studies have revealed associations between the serum levels of proinflammatory cytokines such as IL-1b, IL-6, and TNF alpha, which all increase the risk of dementia and AD.^9^,^10^,^11^,^12^,^13^,^14^,^15^,^16^,^17^,^18^,^19^,^20^,^21^,^22^,^23^,^24^,^25^,^26^,^27^,^28^,^29^,^30^,^31^. An animal model study revealed that OA with peripheral inflammation triggers neuroinflammation and subsequently induces AD pathogenesis^8^. Therefore, we hypothesised that inflammation caused by OA is one of the possible mechanisms of dementia development.

Another possible mechanism through which patients with OA develop dementia is related to the influence of the depression symptoms of OA patients. A meta-analysis reported that patients with a history of depression were highly likely to be diagnosed with dementia later in life^32^. The prevalence of depression is 18% in patients with arthritis aged older than 45 years in the United States^33^. Another article stated that approximately 19% of patients with OA experience moderate-to-severe depression, reporting that chronic pain and impairment of daily activities caused by OA play a crucial role in depression^34^. A previous study revealed that knee joint replacement can improve both mental health and knee function, particularly in patients with OA and a poor preoperative mental health status^35^. Therefore, we infer that a poor mental status caused by OA is among the risk factors contributing to dementia development.

This study had several limitations that must be addressed. First, the diagnosis of dementia and medical comorbidities was determined using ICD-9-CM codes from the NHRID; however, no information on the accuracy of these codes is available. To reduce the bias caused by the incorrect use of codes, we included only patients who received 5 consecutive diagnoses of OA in outpatient clinics or a primary diagnosis of OA during hospitalisation. Second, information on daily physical activity, individual behaviour, and psychological status is not recorded in the NHIRD. These factors are crucial in the development of dementia; however, quantifying these parameters in such a large database is challenging. Finally, the NHIRD does not include data on body weight or the severity and duration of OA. We controlled for age as one of the confounding factors in both cohorts, potentially eliminating this bias because OA is a degenerative disease.

## Conclusion

Patients with OA are at a higher risk for dementia than are those without OA. Impaired daily physical activity, OA-induced inflammatory processes, and depression are possible mechanisms of dementia development. Multidisciplinary interventions that involve controlling pain and symptoms of depression, preventing the decline of physical function, and encouraging participation in leisure activities can be used for OA patients to prevent the development dementia. In the future, studies can focus on the prevention of dementia by controlling the progression of OA symptoms.

## Author Contributions

S.W.H. conceptualised and designed the study and drafted the article; S.W.H. and H.W.L. analysed and interpreted the data; S.W.H. and C.D.L. critically revised the article for important intellectual content; T.H.L. and H.W.L. provided final approval of the article; L.C.C. provided study materials and patients; H.W.L. offered statistical expertise; and W.T.W. provided administrative, technical, and logistical support.

## Additional Information

**How to cite this article**: Huang, S.-W. *et al.* Osteoarthritis Increases the Risk of Dementia: A Nationwide Cohort Study in Taiwan. *Sci. Rep.* **5**, 10145; doi: 10.1038/srep10145 (2015).

## Figures and Tables

**Table 1:** Demographic characteristics and comorbidities for patients in the OA and non-OA cohorts from 2004 to 2007.

**Baseline variable**	**OA cohort n = 35,149**		**Non-OA cohort n = 70, 298**		**P** **value**
	**No ( %)**		**No ( %)**		
**Characteristics**					
Sex					
Female	22216	63.2	44432	63.2	
Male	12933	36.8	25866	36.8	
Age (years)					
18–30	503	1.4	1006	1.4	
31–40	1024	2.9	2048	2.9	
41–50	3599	10.2	7198	10.2	
51–60	7162	20.4	14324	20.4	
61–70	10430	29.7	20860	29.7	
>70	12431	35.4	24862	35.4	
Autoimmune disease (RA, SLE)					<0.001
Yes	3205	9.1	2367	3.4	
No	31944	90.9	67931	96.6	
DM					<0.001
Yes	9997	28.4	16347	23.3	
No	25152	71.6	53951	76.7	
Hypertension					<0.001
Yes	21475	61.1	34734	49.4	
No	13674	38.9	35564	50.6	
Hyperlipidaemia					<0.001
Yes	13417	38.2	17614	25.1	
No	21732	61.8	52684	74.9	
Coronary heart disease					<0.001
Yes	11410	32.5	15135	21.5	
No	23739	67.5	55163	78.5	
COPD					<0.001
Yes	13359	38.0	18728	26.6	
No	21790	62.0	51570	73.4	
Stroke					<0.001
Yes	7156	20.4	11549	16.4	
No	27993	79.6	58749	83.6	
Parkinson’s disease					<0.001
Yes	1283	3.7	1745	2.5	
No	33866	96.3	68553	97.5	

RA: rheumatoid arthritis; SLE: systemic lupus erythematosus; DM: diabetes mellitus; COPD: chronic obstructive pulmonary disease

**Table 2:** Crude and adjusted hazard ratios (HR) for dementia between patients in the OA and non-OA cohorts during the 4-year follow-up period, starting from the index date of ambulatory care visit (n = 105,447).

Presence of dementia	Non-OA patients	OA patients
4-year follow-up period		
Yes/Total (%)	650/70298 (0.92)	421/35149 (1.19)
Person-years	442020	193732
Incidence per 10,000 person-years	14.7	21.7
Crude HR (95% CI)	1.00	1.33* (1.17–1.50)
Adjusted^a^ HR (95% CI)	1.00	1.25* (1.10–1.43)

^a^Adjustments were made for age, sex, autoimmune disease, DM, hypertension, hyperlipidaemia, coronary heart disease, stroke, COPD, and Parkinson’s disease.*indicates P < 0.001

**Table 3:** Adjusted HRs for dementia in the OA and non-OA cohorts determined using the frailty model.

**Presence of dementia during the 4-year follow-up period**			
	**Crude HR (95% CI)**	**Adjusted HR (95% CI)**	**P** **value for adjusted HR**
OA	1.32 (1.17–1.49)	1.21 (1.07–1.38)	0.002
Age (year)	1.09 (1.08–1.10)	1.08 (1.07–1.09)	<0.001
Sex (M)	0.90 (0.79–1.02)	0.73 (0.65–0.83)	<0.001
Autoimmune disease	1.22 (0.95–1.55)	1.14(0.90–1.46)	0.299
DM	1.73 (1.53–1.96)	1.25 (1.10–1.42)	<0.001
Hypertension	2.57 (2.24–2.95)	1.06 (0.92–1.23)	0.381
Hyperlipidaemia	0.98 (0.86–1.12)	0.92 (0.80–1.05)	0.218
Coronary heart disease	1.85 (1.63–2.09)	0.94 (0.82–1.07)	0.342
Parkinson’s disease	8.44 (7.26–9.82)	2.98 (2.54–3.49)	<0.001
COPD	2.15 (1.91–2.42)	1.27 (1.12–1.44)	<0.001
Stroke	6.58 (5.83–7.43)	3.14 (2.75–3.58)	<0.001

To adjust for the hospital cluster random effect, we proposed the frailty model.
